# Impact of Helminth Infection on the Clinical and Microbiological Presentation of Chagas Diseases in Chronically Infected Patients

**DOI:** 10.1371/journal.pntd.0004663

**Published:** 2016-04-26

**Authors:** Fernando Salvador, Elena Sulleiro, Adrián Sánchez-Montalvá, Mónica Martínez-Gallo, Eugenia Carrillo, Israel Molina

**Affiliations:** 1 Department of Infectious Diseases, Vall d’Hebron University Hospital, Universitat Autònoma de Barcelona, PROSICS Barcelona, Barcelona, Spain; 2 Department of Microbiology, Vall d’Hebron University Hospital, PROSICS Barcelona, Barcelona, Spain; 3 Immunology Division, Vall d’Hebron University Hospital, Barcelona, Spain; 4 WHO Collaborating Centre for Leishmaniasis, Centro Nacional de Microbiología, Instituto de Salud Carlos III, Madrid, Spain; George Washington University, UNITED STATES

## Abstract

**Background:**

Helminth infections are highly prevalent in tropical and subtropical countries, coexisting in Chagas disease endemic areas. Helminth infections in humans may modulate the host immune system, changing the Th1/Th2 polarization. This immunological disturbance could modify the immune response to other infections. The aim of this study is to evaluate the relationship between clinical, microbiological and epidemiological characteristics of Chagas disease patients, with the presence of helminth infection.

**Methods:**

A prospective observational study was conducted at Vall d’Hebron University Hospital (Barcelona, Spain). Inclusion criteria were: age over 18 years, diagnosis of Chagas disease, and not having received specific treatment for Chagas disease previously to the inclusion. The study protocol included Chagas disease assessment (cardiac and digestive evaluation, detection of *T*. *cruzi* DNA measured by PCR in peripheral blood), and helminth infection diagnosis (detection of IgG anti-*Strongyloides stercoralis* by ELISA, microscopic examination of stool samples from three different days, and specific faecal culture for *S*. *stercoralis* larvae).

**Results:**

Overall, 65 patients were included, median age was 38 years, 75.4% were women and most of them came from Bolivia. Cardiac and digestive involvement was present in 18.5% and 27.7% of patients respectively. *T*. *cruzi* PCR was positive in 28 (43.1%) patients. Helminth infection was diagnosed in 12 (18.5%) patients. No differences were observed in clinical and epidemiological characteristics between patients with and without helminth infection. Nevertheless, the proportion of patients with positive *T*. *cruzi* PCR was higher among patients with helminth infection compared with patients without helminth infection (75% vs 35.8%, p = 0.021).

**Conclusions:**

We observed a high prevalence of *S*. *stercoralis* infection among chronic Chagas disease patients attended in our tropical medicine unit. Strongyloidiasis was associated with significantly higher proportion of positive *T*. *cruzi* RT-PCR determined in peripheral blood.

## Author Summary

Helminth infections (viz. *Strongyloides stercoralis*, hookworms, *Ascaris lumbricoides*, *Trichuris trichiura*) are highly prevalent in tropical and subtropical areas, and some of these infections may persist in the human host for many years after leaving the endemic area. It is known that helminth infection in humans may modulate the host immune system. This immunological disturbance could modify the immune response to other infections or the antibody production after vaccination. We prospectively studied a group of patients with chronic Chagas disease, with the aim of evaluate the impact of helminth co-infection in the clinical manifestations and microbiological features of Chagas disease. We observed a high prevalence of helminth infection (mostly due to *S*. *stercoralis* infection) among chronic Chagas disease patients attended in our tropical medicine unit. Strongyloidiasis was associated with significantly higher proportion of positive *T*. *cruzi* RT-PCR determined in peripheral blood. These data increase the scarce available information to understand the role of PCR techniques in the management of Chagas disease patients. Further studies are needed to deepen and confirm this interesting relationship.

## Introduction

Chagas disease is a parasitic infection caused by the hemoflagellated protozoan *Trypanosoma cruzi*. Chagas disease is an endemic disease of Latin America affecting rural and poor population; nevertheless, progressive urbanization and the increase of population mobility during last decades, have made Chagas disease an urban and global disease outside endemic countries: mainly in United States and Spain [1, 2].

After the acute phase of the infection, a subsequent usually asymptomatic chronic stage (or indeterminate phase) takes place during years; after 20–30 years, up to a 30–40% of patients will develop the symptomatic chronic phase, with cardiac and/or digestive involvement [2]. Chagas disease diagnosis in the chronic phase is based on serological tests. Due to the low parasitaemia in this phase, classical direct parasitological tests (microhematocrit, hemoculture, xenodiagnose) are usually negative [3]. Nevertheless, more sensitive tests such as the polymerase chain reaction (PCR) have being developed [4]. The percentage of positive *T*. *cruzi* PCR in peripheral blood in patients with Chagas disease in the chronic phase highly varies depending on the study: it ranges from 80% to 90% in studies performed in endemic countries, and is lower in non-endemic countries, ranging from 28% to 66% [5–10]. *T*. *cruzi* PCR is not routinely performed in the management of chronic Chagas disease patients, but is becoming very useful in specific situations, such as follow-up in immunosuppressed patients in order to detect reactivation or in clinical trials to detect treatment failures [11–13]. However the role of the *T cruzi* PCR in the chronic phase of Chagas disease needs to be defined.

Helminth infections (viz. *Strongyloides stercoralis*, *Necator americanus*, *Ancylostoma duodenale*, *Ascaris lumbricoides*, *Trichuris trichiura*) are highly prevalent in tropical and subtropical areas, coexisting in Chagas disease endemic areas, and some of these infections may persist in the human host for many years after leaving the endemic area [14]. It is known that helminth infection in humans may modulate the host immune system, changing the Th1/Th2 polarization. This immunological disturbance could modify the immune response to other infections or the antibody production after vaccination [15, 16].

The aim of the present study is to evaluate the relationship between clinical and epidemiological characteristics of chronic Chagas disease patients, with the presence of helminth infection.

## Materials and Methods

### Ethics statement

The study protocol was approved by the Ethical Review Board of the Vall d’Hebron University Hospital (Barcelona, Spain), and written informed consent was obtained from all patients. Procedures were performed in accordance with the ethical standards laid down in the Declaration of Helsinki as revised in 2000.

### Study protocol

This is a prospective observational study performed at the Infectious Diseases Department of the Vall d’Hebron University Hospital, a tertiary hospital included in the International Health Program of the Catalan Health Institute (PROSICS Barcelona, Spain), from March 2014 to February 2015. All adults (over 18 years old) with recently diagnosis of Chagas disease in the chronic or indeterminate form attended during the study period were offered to participate. Exclusion criteria included: previous treatment for Chagas disease or helminth infections, pregnancy or immunosuppression.

Diagnosis of Chagas disease was performed through two positive different serological tests according to WHO recommendations [17]: an enzyme-linked immunosorbent assay (ELISA) with recombinant antigen (Bioelisa Chagas, Biokit, Lliçà d’Amunt, Spain), and an ELISA with crude antigen (Ortho *T*.*cruzi* ELISA, Johnson & Johnson, High Wycombe, United Kingdom). Cardiac and digestive involvement was assessed through a clinical symptoms questionnaire, physical examination, 12-lead electrocardiography, chest radiography, and barium enema. Patients were stratified according to the clinical Kuschnir classification for cardiac involvement assessment [18]. Pathologic barium enema was defined by dolichocolon or sigmoid diameter > 6cm (megacolon) [19]. A real time PCR (RT-PCR) to detect *T*. *cruzi* DNA in peripheral blood was performed in all patients according to the method described by Piron *et* al [20].

For helminth infection diagnosis, microscopic examination of stool samples from three different days after concentration techniques using Ritchie’s formalin-ether technique were performed in all patients. A faecal culture for *S*. *stercoralis* larvae detection (charcoal culture) was also performed. Moreover, blood cell count to detect presence of eosinophilia (defined as ≥500 cells/mm^3^ and/or ≥7%), and detection of serum IgG anti-*S*. *stercoralis* by ELISA (SciMedx Corporation, Denville, NJ, United States) were conducted.

Definition of helminth infection included: confirmed infections through direct observation, and probable infection (presence of eosinophilia and positive *S*. *stercoralis* serology in the absence of other causes of eosinophilia).

### Statistical analysis

Categorical data are presented as absolute numbers and proportions, and continuous variables are expressed as medians and ranges. The χ^2^ test or Fisher exact test, when appropriate, was used to compare the distribution of categorical variables, and the Mann-Whitney U test for continuous variables. Results were considered statistically significant if the 2-tailed P value was <0.05. SPSS software for Windows (Version 19.0; SPSS Inc, Chicago, IL, USA) was used for statistical analyses.

## Results

Overall, 72 patients were included during the study period. Six patients were excluded because they did not complete the study protocol; therefore, 66 patients were analyzed. The median age of patients was 38 (18–67) years, and 50 (75.8%) were women. The vast majority came from Bolivia (64 patients, 97%) and, at the time of the first visit, the median duration of residence in our country was 9 (1–14) years, and 41 (62.1%) patients had traveled again to their countries after arriving to Spain (most of them spent less than 2 months in their countries, and stayed in an urban setting). Cardiac involvement was diagnosed in 12 (18.2%) patients (nine patients in the stage I, and three patients in the stage II of the Kushnir classification respectively). Eighteen (27.3%) patients presented abnormalities in the barium enema: 16 patients with dolichocolon, and 2 patients with megacolon. At the time of Chagas disease diagnosis, *T*. *cruzi* RT-PCR in peripheral blood was positive in 28 (42.4%) patients.

Helminth infection was diagnosed in 12 (18.2%) patients: two patients with confirmed infection (one patient with *S*. *stercoralis* and another patient with *Hymenolepis nana*), and 10 patients with probable infection. Patients with helminth infection had a median eosinophil cell count of 500 (100–1200) cells/mm^3^. Table 1 shows other protozoan parasites observed in the microscopic examination of stool samples. When comparing main clinical and epidemiological characteristics between patients with and without helminth infection, no differences were observed (Table 2). Nevertheless, the percentage of patients with positive *T*. *cruzi* RT-PCR was higher in patients with helminth infection compared with those without helminth infection (75% versus 35.2%, p = 0.021).

**Table 1:** Protozoan parasites observed in stool samples of Chagas disease patients.

Parasites isolated	Number of patients (n = 66)
**Parasites of uncertain significance**	
*Blastocystis hominis*	23 (34.8%)
*Dientamoeba fragilis*	7 (10.6%)
**Non pathogenic**	
*Entamoeba coli*	6 (9.1%)
*Entamoeba* sp	3 (4.5%)
*Iodamoeba butschlii*	3 (4.5%)
*Endolimax nana*	2 (3%)

**NOTE.** Data are reported as number (%) of patients.

**Table 2:** Main clinical and epidemiological characteristics of Chagas disease patients visited at Vall d’Hebron University Hospital from March 2014 to February 2015.

	Overall (n = 66)	Patients with helminth infection (n = 12)	Patients without helminth infection (n = 54)	*P* value
Age, years	38 (18–67)	41 (28–65)	38 (18–67)	0.247
Gender, male	16 (24.2%)	5 (41.7%)	11 (20.4%)	0.145
Time of residence in our country, years	9 (1–14)	9.5 (1–14)	9 (1–14)	0.967
Cardiac involvement	12 (18.2%)	3 (25%)	9 (16.7%)	0.376
Digestive involvement	18 (27.3%)	3 (25%)	15 (27.8%)	1
Positive *T*. *cruzi* RT-PCR	28 (42.4%)	9 (75%)	19 (35.2%)	0.021

**NOTE.** Data are reported as number (%) of patients or median (range).

## Discussion

We prospectively studied 66 adult patients with Chagas disease to evaluate the relationship between microbiological, clinical and epidemiological characteristics with the presence of helminth infection. Positive *T*. *cruzi* RT-PCR was more frequent in patients with helminth infection compared with those without helminth infection.

Although the study was carried out in a limited group of Chagas disease patients, clinical and epidemiological characteristics found in the study population were similar to those found in larger studies performed in non-endemic countries: most of them coming from Bolivia, young people, majority of women, and low prevalence of cardiac and digestive involvement [2, 8–10]. Therefore, our study population is representative of the Chagas disease population diagnosed and treated in non-endemic areas.

Helminth infection has been diagnosed in 18.2% of the study population, being strongyloidiasis the most frequent infection (all except from one). The *S*. *stercoralis* infection predominance was rather expected, since the median time of residence in Spain in our population was 9 years, thus decreasing the probability of other helminth infections such as *Ascaris lumbricoides*, hookworms or *Trichuris trichiura*. *S*. *stercoralis* is distributed worldwide, being more frequent in tropical and subtropical areas. High prevalence has been found in Latin American countries where Chagas disease is also endemic, hence co-infection is supposed to be high in this area [21]. Scarce information about the prevalence of strongyloidiasis in Bolivia is available, and it is centered in at risk groups [22]. A study published by Ramos *et al* showed a *S*. *stercoralis* seroprevalence of 44.4% among Bolivian immigrants living in Spain [23]. Although our study was not focused on intestinal protozoa, it is important to note the high prevalence of *Blastocystis hominis* and *Dientamoeba fragilis* infections (34.8% and 10.6% respectively) observed in our study population; despite their pathogenicity remains uncertain and controversial, the presence of these parasites may be used as a marker of potential exposure to other pathogenic parasites.

When comparing epidemiological, clinical and microbiological characteristics between patients with and without helminth infection, the first group had statistically significant higher proportion of positive *T*. *cruzi* RT-PCR in peripheral blood than the second group (75% and 35.2% respectively). To our knowledge, no previous study has addressed the possible implications of Chagas disease and helminth co-infection in humans. Nevertheless, some interesting studies in animal model have been published with similar findings. Monteiro *et al* described higher prevalence of *T*.*cruzi*-positive blood cultures in golden lion tamarins infected with *T*. *cruzi* when they were co-infected with intestinal helminths of the Trichostrongylidae family, which is coherent with the results obtained in our study [24]. Another study performed with *T*. *cruzi* infected mice went in depth in this relationship between helminth infection and *T*. *cruzi* parasitaemia: no differences in the parasitaemia were found between non co-infected and early co-infected mice (the *T*. *cruzi* infection took place 2–4 weeks after *Taenia crassiceps* infection), however, late co-infected mice (the *T*. *cruzi* infection took place 8–12 weeks after *Taenia crassiceps* infection, when a predominant Th2-type cytokine response is expected) showed significantly higher parasitaemia compared with non co-infected and early co-infected mice [25].

*Strongyloides* spp infection in the murine model induces a Th2 response and regulatory cytokine induction (IL-10), leading to a suppression of pro-inflammatory cytokines and diminishing Th1 response [26]. These pro-inflammatory cytokines (Th1 response) are present in the acute phase of Chagas disease [27]. Thus, co-infection with different parasites may result in complex interactions, which may lead to altered immunological responses of the host.

The relationship between positive *T*.*cruzi* RT-PCR in peripheral blood and helminth infection (mostly strongyloidiasis) found in this study provides highly relevant data to better understand the role of the PCR in the management of Chagas disease patients. Helminth infection could increase the probability of having a positive *T*. *cruzi* PCR. Given that current clinical trials that evaluate treatment efficacy in Chagas disease are based on the positivity of *T*. *cruzi* PCR, this fact may be relevant. [13, 28]. Further studies are needed to evaluate the impact of treating the helminth infection on the positivity of *T*. *cruzi* PCR.

Another issue that has to be taken into account is that almost all patients in our study came from Bolivia. The geographical distribution of the different *T*. *cruzi* discrete typing units (DTUs) differs from country to country, which may have impact in the clinical presentation or in the proportion of patients with positive *T*. *cruzi* PCR in peripheral blood [29].

This study has some limitations. First of all, as we have mentioned previously, the study has been performed with a relatively small number of patients; nevertheless, the study population is representative of Chagas disease patients attended in Spanish tropical medicine units. Secondly, the diagnosis of strongyloidiasis has relied in serological tests in most of the cases. Although serology is not the gold standard for the *S*.*stercoralis* infection diagnosis, previous studies have demonstrated its usefulness [30]. New tests based on molecular biology such as PCR could increase the accuracy of helminth infection diagnosis. Finally, *T*. *cruzi* PCR was determined only at one point, which may underestimate the kinetics of the parasite.

In summary, we observed a high prevalence of *S*. *stercoralis* infection among chronic Chagas disease patients attended in our tropical medicine unit. Strongyloidiasis was associated with significantly higher proportion of positive *T*. *cruzi* RT-PCR determined in peripheral blood. These data increase the scarce available information to understand the role of PCR techniques in the management of Chagas disease patients. Further studies are needed to deepen and confirm this interesting relationship.

## Supporting Information

S1 Checklist STROBE Checklist. (DOCX)
